# Learning from the Brazilian Community Health Worker Model in North Wales

**DOI:** 10.1186/1744-8603-9-25

**Published:** 2013-06-11

**Authors:** Christopher David Johnson, Jane Noyes, Andy Haines, Kathrin Thomas, Chris Stockport, Antonio Neves Ribas, Matthew Harris

**Affiliations:** 1 Public Health Wales, Preswylfa, Mold, Flintshire CH71 PZ, UK; 2 School of Healthcare Sciences, Bangor University, Bangor, Gwynedd LL57 2EF, UK; 3 Department of Social and Environmental Health Research, London School of Hygiene and Tropical Medicine, London WC1H 9SH, UK; 4 Department of Population Health, London School of Hygiene and Tropical Medicine, 15-17 Tavistock Place, London WC1H 9SH, UK; 5 Primary & Community Services, Betsi Cadwaladr University Health Board, Preswylfa, Mold, Flintshire CH71 PZ, UK; 6 Health Care Secretariat, Ministry of Health, Esplanada dos Ministérios, Bloco G, Brasília, DF, Brazil; 7 Department of Primary Care and Public Health, Imperial College London, 3rd Floor, Reynolds Building, Charing Cross Campus, St Dunstan Road, London W6 8RP, UK

**Keywords:** Community Health Worker, Reverse innovation, Primary care

## Abstract

Health policymakers in many countries are looking at ways of increasing health care coverage by scaling up the deployment of community health workers. In this commentary, we describe the rationale for the UK to learn from Brazil’s scaled-up Community Health Worker primary care strategy, starting with a pilot project in North Wales.

## Background

Health policymakers in many countries are looking at ways of increasing health care coverage by scaling up the deployment of community health workers (CHWs), lay members of the community who are provided with short periods of training, and supervision, to work in primary care settings. Traditionally this has been seen as an option for low and middle income countries with inadequate numbers of trained health professionals. In this commentary, we describe the rationale for the UK to learn from Brazil’s scaled-up CHW primary care strategy, starting with a pilot project in North Wales.

### Brazilian community health worker model – the family health strategy

In 1988 the Brazilian Government created the Sistema Unico de Saúde (SUS) or Unified Health System to provide universal care free at the point of delivery [1]. Part of this universal care programme is the Family Health Strategy – a nationally scaled model of primary care services, each covering geographically defined areas of up to 4000 inhabitants. Primary Care teams are composed of a doctor, a nurse, a nurse auxiliary and at least four Community Health Workers (CHWs), recruited from the local community and who are each responsible for up 750 people (approximately 100-150 households) in their micro-areas. The lay CHWs visit each household in their micro-areas every month regardless of expressed need or demand. No households are excluded and no household is unattended. CHWs are recruited from the neighbourhoods where they are deployed, trained for up to three months and are employed by the municipal health authority. Table 1 shows the wide range of activities that they are expected to deliver in their micro-areas. Consequently they are instruments for both public health action and community empowerment [1], particularly since the model has been scaled to a national level with over 54% of the Brazilian population covered. There are now 257,265 lay CHWs in Brazil. Although uptake of the model has been somewhat slower in large urban centres, where upper middle class populations are served predominantly by private health insurers, the Family Health Strategy is probably the most successful example of primary care reform in the world.

**Table 1:** Activities carried out by CHWs in the Brazilian family health strategy

	**Area**	**Specific activities**
Primary Care	Chronic Disease Management	Supporting adherence to medications for diabetes and hypertension. Early identification of symptoms and signs of chronic disease and referral into the family health unit. Design, organize and deliver group education meetings for individuals with chronic disease e.g. diabetes and hypertension
	Triage	Low level referral support to ensure appropriate use of primary care services. Early identification of clinical problems and referral into the family health unit. Support with appointment booking, call/recall.
	Clinical Care	Use of Integrated Management of Childhood Illness decision support tool to identify children at risk of serious illness
	Social Determinants	Identification of household determinants of ill health and health seeking behaviour
	Vertical Disease Programmes	TB DOTS service support providing daily home visits
	Healthy Pregnancy /Child development	Ensure early booking and healthy lifestyle during pregnancy. Antenatal consultation support. Design, organize and deliver low-risk pregnancy health education groups. Monthly or bimonthly weight measurement in all children less than 2 years of age
Public Health	Screening	Improve uptake in cervical and breast screening through identification of target groups and signposting
	Immunizations	Identification of children with incomplete vaccine schedules and referral into the GP practice
	Breastfeeding support	Peer support for breastfeeding providing one-to-one advice in the household. Design, organize and deliver group education meetings for breast-feeding mothers
	Health Promotion	Delivery directly into the household of healthy lifestyle messages such as stop smoking, hand hygiene, weight loss, healthy diet and physical exercise with referral and signposting where appropriate
	Health Protection	Contact tracing for common infectious diseases e.g. typhoid fever. Household risk assessment and advice for vector-transmitted disease e.g. dengue fever
	Household data collection	Monthly collection and update of basic household data on occupancy, socio-demographic data, education levels, occupation, deprivation and predominant health issue including smoking and alcohol use of every member in each household
Community	Health education groups	Includes but not limited to breastfeeding support, diabetes, hypertension, low-risk pregnancy and immunizations health education groups
	Planning and performance	Engaging with the community to ensure health services are satisfactory and appropriate in their design and delivery
	Community Liaison	Acting as a liaison between the health service and community leaders. Acting as a community leader

Over the last 20 years since implementation there have been significant reductions in infant mortality [2], hospitalizations due to primary care sensitive conditions [3], improvements in screening uptake, improvements in breast-feeding uptake, antenatal care, mental health problems and immunization coverage [4] which can be plausibly attributed to the programme, and it has led to pro-poor improvements in health equity, closing the gap between the rich and poor [5]. The experience of the Brazilian Family Health Strategy, shown to be a cost-effective model [6] with high levels of user satisfaction [7], adds to an increasing array of evidence in support of using lay CHWs as a cadre within primary care services [8,9], not only in low and middle-income countries but also in high-income settings [10] (Table 1).

### Reverse innovation in North Wales

North Wales has a population of around 676,000 spread across 6500 sq Km [11], and has significant health inequalities [12] largely as a result of health improvements being distributed unevenly between the most deprived and least deprived areas. There are significant gender- and deprivation-related differences in life expectancy [12] and uptake of immunization and screening programmes remain stubbornly below national averages [11].

The Betsi Cadwaladr University Health Board (BCUHB) is responsible for providing a full range of primary, community, mental health and acute hospital services for North Wales [13]. Inspired by the successes of the Brazilian Family Health Strategy, BCUHB and Bangor University have teamed up with researchers at the London School of Hygiene and Tropical Medicine and Imperial College London to investigate whether successes from the Brazilian model can be translated into improvements in community health services in some of the most challenging areas of Wales. Although there are many different CHW models to learn from, the Brazilian approach, (universal, comprehensive cradle-to-grave service and integrated into primary care) has proven scalability, and by working across traditional boundaries offers potential added value in North Wales. The universal, comprehensive and integrated role of the Brazilian CHWs contrasts with the existing vertical, targeted and fragmented approaches currently deployed in North Wales, and the rest of the UK. Table 2 shows the diverse array of community-based professionals currently deployed to deliver specific activities in North Wales. Initial interest in this proposal has been high, especially amongst local and regional health and social care policy makers, doctors and nurses working in primary and community care services.

**Table 2:** List of community services available across North Wales

**Pregnancy**	**Children**	**Adult**	**Social**	**3**^**rd **^**Sector teams**
Community midwife	Social workers Safeguarding teams	Specialist nurses: Tissue viability; Alzheimer’s; Parkinson’s; Diabetes; Stroke; Heart failure; Respiratory; Oxygen assessment; Home ventilation; Immunisation Practice Nurses Vulnerable group teams Crisis intervention teams Early discharge teams Contact tracing	Home carers Education psychologists Youth workers Language and play support workers (LAP) Numbers and play support workers (NAP) Families First Intensive family support services Financial advisors Debt and benefit maximization teams Peer parents Community support officers Communities First workers	Flying start Health promotion worker
Health visitor Midwifery support	Child mental health teams			User involvement Counselling services – Bernardo’s / NSPCC / NCH Action 4 Children Advocacy services Homestart Breastfeeding support
Specialist nurses: Learning disability; Substance misuse; Community Psychiatry Specialist midwife Drug and alcohol team	Community paediatrician			
	Childcare support workers			
	Specialist nurses: Community Diana; Neonatal; Diabetes; Nursery; Art therapy; Specialist Behavioural; Occupational therapy; Youth justice service			
Asylum specialist				
	Play workers Speech and Language therapists			

The potential shift from the current reactive, curative services targeted to those with expressed need or demand, to proactive, holistic whole-household based health promotion could be a sustainable solution to escalating workforce costs, empower patients and ensure more appropriate access to relevant services. Although, some eventual task-shifting would be required, within North Wales this could lead to new opportunities for cost-saving and in the long run, the improved integration of health and social care services [Harris M, Hughes M, Johnson C, Noyes J, Roberts A and Stockport C: *Learning from Community Health Workers in Brazil: post-visit report.* BCUHB/Bangor University/Imperial College; 2013, unpublished report). As a potentially ‘disruptive innovation‘, where new sets of values eventually overtakes current practice such a radical proposition requires careful change management.

Consequently, the first step to be undertaken in North Wales will be a small pilot study to test the feasibility and acceptability of this approach, as communities in Wales have a different history, experience, and expectations of health care services than families in Brazil. We will assess the feasibility, acceptability and potential impact of lay CHWs, integrated into primary care services, delivering comprehensive, cradle-to-grave, household-based health promotion advice, to defined micro-areas of around 80 households, and to optimize the role and its evaluation sufficiently to take to full cluster Randomized Controlled Trial. There will be four CHWs (80 households each) to be employed in the study, integrated into GP practices, and recruited from the area where they will be working. They will support adherence to medications and appropriate diet for diabetes and hypertension and identify complications; identify symptoms and signs of dementia; provide smoking cessation and alcohol use reduction advice; provide advice on healthy diet and physical exercise across the lifespan; identify those eligible for cancer screening and immunization uptake; provide basic contraceptive and safe sex advice; encourage Chlamydia screening uptake in target populations; support early booking and referral for new pregnancies and provide basic post-pregnancy contraception advice including basic breast-feeding support; and monitor the growth and development in the under 2s.

We will ascertain the acceptability of the lay CHWs from the patient, provider and policy-maker perspectives and the programme theory of what works, for whom and why, based on realist evaluation of the CHWs’ role. We will estimate the relative effectiveness of the lay CHWs on uptake of immunization and screening services; detection of chronic diseases; and giving health promotion and lifestyle advice. We will identify whether the CHW role is likely to offer a return on investment sufficient to justify a rigorous trial. The proposal is a collaboration between researchers and implementers at Imperial College, Bangor University, Betsi Cadwaladr University Health Board, Public Health Wales, and the London School of Hygiene and Tropical Medicine.

The Brazilian model has grown within an evolving health system however the challenge in North Wales is to introduce community health workers into an already crowded and established health and social care space requiring careful task shifting to create a system that adds value, rather than complexity. Additionally, North Wales is a bilingual area, with first language Welsh speaking being especially strong in Western areas. Incorporating language sensitivity and providing services in the language of preference, will present important acceptability challenges.

If successful, the next step will be an adequately powered randomised cluster controlled trial to detect changes in individual and population health outcomes, with an embedded health economic analysis and process evaluation to determine cost-effectiveness and barriers and facilitators to wide scale implementation. The study will provide opportunities for novel methodological development in trial design and economic evaluation and implementation processes to better understand the critical success factors for successful innovation of complex interventions adapted from experience in a different setting within a ‘realist’ context [Department of Health: *Evaluation of the Brazil-UK Memorandum of Understanding.* United Kingdom: United Kingdom Department of Health; 2013. unpublished report].

As with any health service change, innovations are usually met with resistance and entrenched interests. However, the North Wales intention to trial the Brazilian CHW model, even at a very small scale initially, has exposed prejudices peculiar to a reverse innovation process. Serious doubt, for example, has been expressed by some local stakeholders about what could possibly be learnt from a ‘developing country like Brazil’ and that the prospects of visiting Brazil might be the dominant motivation for the interest of some collaborators. Concern has also been expressed over the cultural differences between Brazil and Wales, specifically that Brazilians might exhibit health-seeking behaviour more amenable to a CHW approach or that communities are inherently more cohesive in Brazil. We note, however, that Brazil overcame such barriers and that until this approach is tried and tested it cannot be assumed how the North Wales patient population will react to it. Over and above the challenges of health service change, it is clear that learning from an emerging economy adds an additional layer of complexity that requires special, tactful and patient management.

The broader collaborative relationship developing between the UK and Brazilian Ministries of Health has been an important lever to foster engagement within Wales. It is specifically the reciprocal learning element, somewhat unusual in North-South collaborations [14] that has strengthened the bilateral relationship. The broader policy context in the UK is also an important lever. There is increasing emphasis on de-professionalising care, bringing care back into the community, and empowering communities to take control of their healthcare needs [15,16]. The fundamental drivers have been to reduce healthcare costs arising from multiple fragmented and duplicated services, and an over-reliance on secondary care service utilization. In financially constrained times, there is pressure to streamline services, identify efficiencies, and improve communication which offers fertile ground for innovation. The recently published WHO guidelines, based on Cochrane systematic reviews, show task shifting generally and community health workers specifically can better serve communities, are generally welcomed by the local people, and deliver care more effectively, efficiently, equitably and locally than many existing services [10]. Now that the WHO recommends task shifting of key functions from expensive health professionals to other less expensive cadres of staff including to lay community health workers, not only in resource-poor settings but in all member states [10], there is a policy imperative to learn from countries that have developed lean solutions to their health care delivery problems, such as Brazil. Both Brazil and the UK are experiencing an epidemiological transition (aging population, rising levels of chronic disease and obesity) and so their contexts are actually becoming increasingly similar. The North Wales reverse innovation activity could potentially be very timely for the UK more broadly [17,18].

## Conclusion

Within Western industrialised countries there is sometimes scepticism about what we have to learn from the experience of emerging economies. Traditionally there has been the assumption that health systems developed in high income countries should be emulated by countries with fewer resources. However health systems in many industrialised states may be unsustainable and can learn from the rapid scale up of innovative delivery approaches in some low and middle income countries Notable achievements in public health outcomes in emerging economies that have been realised through relatively low cost, simple, and effectively implemented and scaled up interventions provide novel opportunities to explore the feasibility of reverse innovation interventions and establish if similar health gains can be achieved in different contexts. With economic recession or stagnation now firmly gripping many industrialised countries, learning from emerging economies will become increasingly necessary.

## Abbreviations

BCUHB: Betsi Cadwaladr University Health Board; CHW: Community health worker; SUS: Sistema Unico de Saúde; WHO: World Health Organisation.

## Competing interests

The authors declare that they have no competing interests.

## Authors’ contributions

CDJ, was the principal author of the commentary. JN, provided comments and revisions to the commentary and is a principal member of the research team for the proposed project. AH, provided comments and revisions to the commentary and is a principal member of the research team for the proposed project. KT, provided comments and revisions to the commentary and is a principal member of the research team for the proposed project. CS, provided comments and revisions to the commentary and is a principal member of the research team for the proposed project. AR, provided comments and revisions to the commentary and is a member of the External Steering committee for the proposed project and MH, provided comments and revisions to the commentary and is the lead researcher for the proposed project. All authors read and approved the final manuscript.
